# Alternative Splicing of RAD6B and Not RAD6A Is Selectively Increased in Melanoma: Identification and Functional Characterization

**DOI:** 10.3390/cells8111375

**Published:** 2019-11-01

**Authors:** Ambikai Gajan, Carly E. Martin, Seongho Kim, Milap Joshi, Sharon K. Michelhaugh, Ido Sloma, Sandeep Mittal, Steven Firestine, Malathy P. V. Shekhar

**Affiliations:** 1 Karmanos Cancer Institute, Detroit, MI 48201, USA; gajana@karmanos.org (A.G.); carly.martin@wayne.edu (C.E.M.); kimse@karmanos.org (S.K.); milap.joshi.5000@gmail.com (M.J.); skmichel@vtc.vt.edu (S.K.M.); sandeepmittal@vt.edu (S.M.); 2 Department of Oncology, Wayne State University School of Medicine, Detroit, MI 48201, USA; 3 Champions Oncology, Rockville, MD 20850, USA; isloma@championsoncology.com; 4 Pharmaceutical Sciences, Wayne State University, Detroit, MI 48201, USA; sfirestine@wayne.edu; 5 Department of Pathology, Wayne State University School of Medicine, Detroit, MI 48201, USA

**Keywords:** melanoma, histone ubiquitination, alternative splicing, exon skipping, whole exome sequencing

## Abstract

Rad6B, a principal component of the translesion synthesis pathway, and activator of canonical Wnt signaling, plays an essential role in cutaneous melanoma development and progression. As Rad6 is encoded by two genes, namely, *UBE2A* (*RAD6A*) and *UBE2B* (*RAD6B*), in humans, we compared their expressions in melanomas and normal melanocytes. While both genes are weakly expressed in normal melanocytes, Rad6B is more robustly expressed in melanoma lines and patient-derived metastatic melanomas than RAD6A. The characterization of RAD6B transcripts revealed coexpression of various splice variants representing truncated or modified functional versions of wild-type RAD6B in melanomas, but not in normal melanocytes. Notably, two RAD6B isoforms with intact catalytic domains, RAD6BΔexon4 and RAD6Bintron5ins, were identified. We confirmed that RAD6BΔexon4 and RAD6Bintron5ins variants are expressed as 14 and 15 kDa proteins, respectively, with functional in vivo ubiquitin conjugating activity. Whole exome sequence analysis of 30 patient-derived melanomas showed RAD6B variants coexpressed with wild-type RAD6B in all samples analyzed, and RAD6Bintron5ins variants were found in half the cases. These variants constitute the majority of the RAD6B transcriptome in contrast to RAD6A, which was predominantly wild-type. The expression of functional RAD6B variants only in melanomas reveals RAD6B’s molecular heterogeneity and its association with melanoma pathogenesis.

## 1. Introduction

Cutaneous melanoma is the most predominant and aggressive form of skin cancer and has the highest burden of somatic genetic alterations. The main genetic drivers are *B-RAF*, *NF1* and *N-RAS*, and melanomas associated with chronically sun-exposed skin show a high UV signature bearing mutation load [1,2,3,4]. Replicative bypass across UV-damaged DNA is dependent upon RAD6-mediated translesion synthesis (TLS), also known as the DNA damage tolerance pathway [5]. Inactivation of RAD6, an ubiquitin conjugating E2 enzyme, or its cognate E3 ligase RAD18 severely impairs error-free and error-prone modes of DNA damage bypass [6]. Activation of TLS requires RAD6/RAD18-induced PCNA monoubiquitination as it enables recruitment of low-fidelity TLS polymerases that have the ability to accommodate DNA sequence distortions such as cyclobutane pyrimidines ensuing from UV-induced damage [7,8,9,10,11]. RAD6/RAD18 is responsible for the monoubiquitination of PCNA in response to all DNA damaging agents that elicit TLS, indicating its broad importance in any circumstance in which the progression of a DNA replication fork is stalled [5].

In humans, RAD6 is encoded by two genes, *UBE2A* (*RAD6A*) and *UBE2B* (*RAD6B*), which are located on chromosomes Xq24-q25 and 5q23-q34, respectively [12,13,14], and share 95% identity in amino acid composition and have similar ubiquitin conjugating activities. Besides the role of RAD6 in TLS, we have demonstrated a relationship between RAD6B and canonical Wnt signaling in melanoma [15,16,17] and in breast cancers [18,19] wherein RAD6B stabilizes β-catenin via 26S proteasome-insensitive K63-linked polyubiquitination, and β-catenin in turn induces *RAD6B* transcription [18,19]. Induction of melanocyte specification and differentiation from neural crest stem cells, and also melanoma promotion, requires Wnt/β-catenin signaling [20]. RAD6 expression is negligible in normal skin; however, increases in its expression correlate with increases in melanocytic proliferation and the disruption of the normal melanocyte to keratinocyte ratio in normal skin [15]. RAD6 is also overexpressed in primary and metastatic melanomas [16], thus indicating a role for RAD6 in early melanoma development and a continued role for RAD6B in melanoma progression and metastasis. 

Changes in transcriptome frequently arise from alternative splicing abnormalities in tumors [21]. Dysregulation or misexpression of alternate spliced isoforms results from mutations or deletions in cis-acting regulatory sequences, or mutations/aberrant expression of splicing trans-factors. An alternative splicing switch may confer a selective advantage to cancer cells as it has been found to prevail during tumorigenesis and correlates with cell proliferation, invasion and metastasis [22,23]. Since melanomas have the greatest mutational burden compared to other cancers, and RAD6B overexpression is implicated in melanoma pathogenesis via its functions in canonical Wnt signaling and TLS, we determined whether RAD6B transcript profiles were altered in melanomas as compared to normal melanocytes. Reflecting the robustness of wild-type RAD6B expression in melanoma cell lines and patient-derived melanoma brain metastases, several alternatively spliced RAD6B transcripts were identified in melanoma lines and clinical melanomas but not in normal melanocytes. We show that recurrent RAD6B isoform switches result from exon skipping events involving exons 2, 3 and/or 4, but not exons 5 or 6. Whereas several of these splice variants are predicted to produce truncated Rad6B due to frameshifts, our analysis also identified functional RAD6B isoforms with intact catalytic domains resulting from exon 4 skipping (RAD6BΔexon4) and another from an intron 5 insertion event (RAD6Bintron5ins). TCGA analysis of RAD6A and RAD6B expressions and copy number variations in melanomas revealed that RAD6B expression is more heterogeneous than RAD6A. Whole exome sequence (WES) analysis of clinical melanomas verified that while RAD6A variants represent only a small portion of the RAD6A transcripts in melanomas, RAD6B variants are co-expressed in 100% of the melanomas analyzed and represent the majority of the RAD6B transcriptome. Since RAD6B isoform switches were not detected in normal melanocytes and common RAD6B isoforms were detected in melanoma samples, our results suggest that the expression of these precise splicing isoforms with functional activity could potentially contribute to melanoma pathogenesis and provide a source for the RAD6B transcript heterogeneity seen in melanoma patients.

## 2. Materials and Methods

### 2.1. Cell Culture and Patient Samples

Normal human epidermal melanocytes HEMa-LP, and also human melanoma A375 and A2058 cells, were purchased from American Type Culture Collection (ATCC, Manassas, VA 20110, USA). Human melanoma M14 cells were obtained from the National Cancer Institute, Bethesda, MD 20892, USA. HEMa-LP cells were maintained in dermal cell basal medium (ATCC) supplemented with the melanocyte growth supplements insulin (5 μg/mL), ascorbic acid (50 μg/mL), L-glutamine (6 mmol/L), epinephrine (1.0 μmol/L), calcium chloride (0.2 mmol/L) and M8 supplement (ATCC). The authenticated cell lines were used within 5–10 passages. Patient-derived metastatic melanoma cell lines 14-089 and 14-108 were generated by dissociation of metastatic brain tumors into single cell suspensions using the GentleMACs Dissociator and Human Tumor Kit (Miltenyi Biotec, San Diego, CA, USA) according to the manufacturer’s protocol. The resulting cultures were grown in Dulbecco’s Modified Eagle Medium (DMEM)/F12 media supplemented with 10% fetal bovine serum, non-essential amino acids and gentamicin (Millipore Sigma, St. Louis, MO, USA) at 37 °C, 5% CO_2_. Malignant melanoma 14–089 was negative for BRAF V600E and V600K, and 14–108 was positive for BRAF V600E. Acquisition and use of clinical samples were approved by the Wayne State University Institutional Review Board and written informed consent was obtained from each patient prior to enrollment (IRB 111610MP2E; Protocol # 1011009008). Patient-derived xenografts were established from 30 primary and/or metastatic melanomas by Champions Oncology, Inc. (Rockville, MD, USA) with written informed consent and approval by the Institutional Animal Care and Use Committee [24]. Details on patient tumor, stage and gender are shown in Supplementary Tables S1 and S2. Research was done according to the Helsinki Declaration and informed patient consent was obtained.

### 2.2. RT-PCR, Subcloning and Sequence Analysis

Total RNA was isolated from normal melanocytes and melanoma lines using Trizol reagent (Invitrogen, Carlsbad, CA, USA). cDNAs were synthesized from 0.75–2 μg of total RNA using Superscript III (Invitrogen), and full-length RAD6B was PCR amplified using forward 5′-TTCAGACTGACCGCGGGGCA-3′ and reverse 5′-AGATTAACAGACCAGTTGTC-3′ primers (Accession # NM_003337). RAD6A was PCR amplified using forward 5′-GGATGGAACATTTAAACTTAC-3′ and reverse 5′-TGCTGGACTATTGGGATTG-3′ primers (Accession # NM_003336), and GAPDH with forward 5′-AAATATGATGACACCAAGAAGG-3′ and reverse 5′-TGAAGTCGGAGGAGACCAC-3′ primers (Accession # NM_002046). RAD6B PCR products were subcloned into the pCR2.1-TOPO vector (Thermo Fisher Scientific, Waltham, MA, USA), and plasmid DNAs purified from transformed colonies were subjected to EcoRI digestion to release the insert. Two to four clones displaying the correct wild-type RAD6B size and all clones showing size variations from the wild-type RAD6B transcript were sequenced in both directions using vector-specific primers.

### 2.3. Expression Analysis

RAD6B splice mutants (RAD6BΔexon4 and RAD6Bintron5ins) were subcloned into the EcoRI site of pCIneo vector (Promega Corp., Madison, WI, USA), and sequences were confirmed by DNA sequencing. Full-length wild-type (WT) RAD6B-pCMVneo (1–2.5 μg) [25] and 3–12 μg RAD6B splice mutants were transiently transfected into COS7 or 293T cells using Metafectine (Biontex Laboratories GmbH, München, Germany), and 48 h post-transfection, total RNA or whole cell lysates were prepared for RT-PCR and Western blot analysis, respectively. To assess the functional activity of RAD6B splice mutants, transfected cultures were treated with 25 μM MG132 approximately 4–5 h prior to cell lysis.

### 2.4. Western Blot Analysis

Whole cell lysates of normal melanocytes, melanoma lines, patient-derived melanoma brain metastases, and RAD6B-transfected cells were prepared as previously described [26]. Aliquots of whole cell lysates containing equivalent amounts of protein were subjected to SDS-PAGE and Western blot analysis with RAD6 antibodies specific to the carboxyl terminus [25] or amino terminus (Bethyl Labs, Inc., Montgomery, TX, USA), and with β-actin (Sigma-Aldrich, St. Louis, MO, USA). To assess the ubiquitin conjugating activity of the RAD6B splice variants, Western blots of MG132 treated and untreated lysates were probed with anti-ubiquityl-histone H2A antibody (Cell Signaling, Danvers, MA, USA).

### 2.5. Homology/Template Modeling of Rad6B Splice Variant Structures

Homology models of the RAD6BΔexon4 and RAD6Bintron5ins variants were created using Swiss-Model from PDB file 2YB6 for RAD6. The models for each protein were exported as PDB files and imported into the Molecular Operating Environment (MOE) for additional analysis. Each model was treated with the QuickPrep function, which automatically corrects for common problems associated with structures in the PDB including missing atoms, geometry conflicts, inappropriate protonation and crystallographic artifacts. Each model was also aligned and superposed with the crystal structure of RAD18 (the cognate E3 ubiquitin ligase for RAD6) bound to RAD6B (2YBF) to examine the binding site of the E3 enzyme.

### 2.6. TCGA Analysis

Gene expression levels and copy number variations (gain) of *RAD6A* (*UBE2A*) and *RAD6B* (*UBE2B*) were extracted from TCGA cutaneous melanoma data through the UCSC Xena Functional Genomics Explorer (UCSC Xena; http://xena.ucsc.edu) along with clinical and pathological information [27]. Gene expression levels were generated from RNAseq-Illumina HiSeq experiments, and copy number variations were obtained after removing germline copy number variations. Both expression levels and copy number variations were log2-transformed. An F-test was used to determine whether the variances of two groups were equal, and then the ratio of RAD6B variance to RAD6A variance (r) was estimated with its associated 95% confidence interval (CI). The associations of RAD6A and RAD6B with tumor stage were evaluated using one-way ANOVA. No correction was made for multiple comparisons. The comparison between RAD6A and RAD6B was performed using the Wilcoxon test for paired samples.

### 2.7. Whole Exome Sequence (WES) Analysis

Whole exome sequencing of early passage patient-derived xenografts (PDX) established from 30 primary/metastatic melanomas was performed by Champions Oncology [24]. WES analysis was performed using an Illumina HiSeq 2500 machine, as pair-end 125 bp reads, following the manufacturer’s protocol with an Agilent v4_XT_51Mb capture kit to reach an average coverage of 100×. Raw reads from each PDX were aligned to a concatenated human + mouse genome (GRC37/hg19 + GRCm38/mm10) using a BWA aligner followed by a filtering process of reads that were not aligned to the human contigs. Local realignment around INDELs and base quality score recalibration was done using the GATK pipeline. Each model’s transcriptome was characterized by RNA-Seq via pair-end 50-bp reads to a 50 million reads spec per sample, and filtering was applied on RNA-Seq data using the STAR aligner. Mouse-filtered FASTq files were used for RSEM analysis of *UBE2A* (*RAD6A*) and *UBE2B* (*RAD6B*) genes and isoform quantifications. The percentages of wild-type RAD6A and RAD6B relative to their corresponding variants were compared using the Wilcoxon test for paired samples.

## 3. Results

### 3.1. The RAD6B Paralog Displays Greater Heterogeneity in Expression Levels and Copy Number Compared to RAD6A in Melanoma Patients

**Figure 1:**
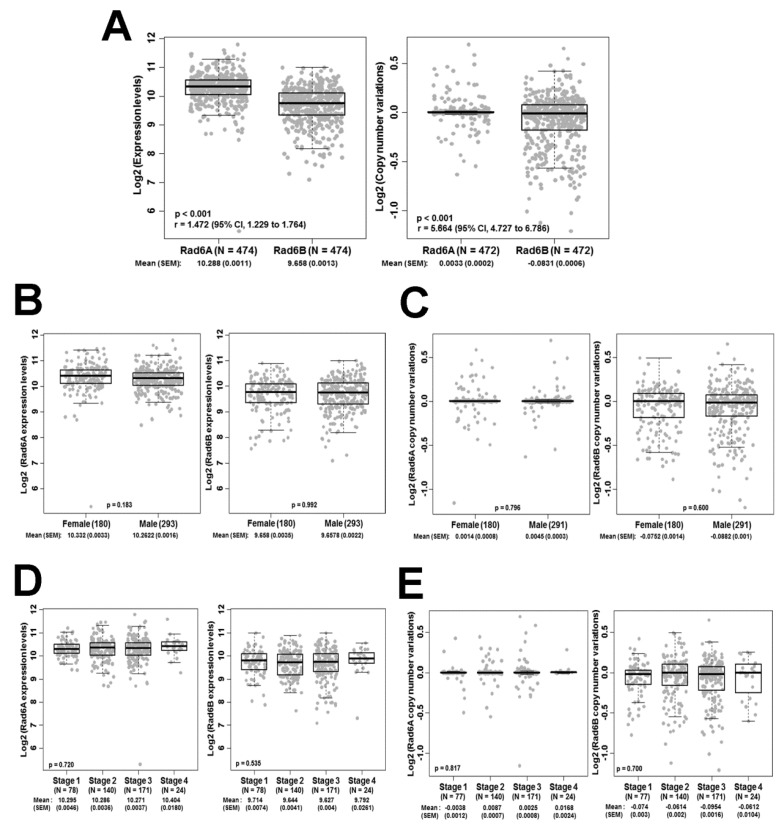
**TCGA analysis of *RAD6A* and *RAD6B* expression levels and copy number variations in cutaneous melanoma patients.** (**A**) The equality of variances between *RAD6A* and *RAD6B* was examined using an F-test, and the ratio of variances is represented by ‘r’ and 95% confidence interval. r > 1 indicates greater heterogeneity for *RAD6B* expression and copy number variations than *RAD6A*. (**B**,**C**). *RAD6A* and *RAD6B* expression (**B**) and copy number variations (**C**) in female and male melanoma patients. (**D**,**E**) *RAD6A* and *RAD6B* expression (**D**) and copy number variations (**E**) with stage of disease. Sample size (N), mean, and standard error of the mean (S.E.M.) are indicated.

We have previously demonstrated that RAD6 expression is weak or negligible in normal skin and that increases in its expression in cutaneous melanomas directly correlate with increases in melanocytic proliferation and transformation [15]. Consistent with these data, we showed that RAD6 is weakly expressed in normal melanocytes and benign nevus but displays overexpression in surgically excised primary and metastatic melanomas as well as in melanoma cell lines [16]. These data implicate an early role for RAD6 in melanoma development and a continued role for RAD6 in melanoma progression and metastatic spread. Since the two RAD6 paralogs, *RAD6A* and *RAD6B* localized on chromosomes Xq24–25 and 5q23–31, respectively, encode for RAD6 proteins with ~95% amino acid sequence identity, we performed TCGA analysis to compare *RAD6A* and *RAD6B* expression levels and copy number alterations in melanoma patients. No significant differences in expression levels and copy number alterations for *RAD6A* and *RAD6B* were observed in a sample population of 474 and 472 melanoma patients, respectively (Figure 1A). *RAD6A* and *RAD6B* expression levels and copy numbers showed no significant differences based on gender (Figure 1B,C). Analysis of *RAD6A* and *RAD6B* transcript levels and copy number at different stages of melanoma progression similarly showed no significant differences (Figure 1D,E). Interestingly, although the mean log2 expression levels between RAD6A and RAD6B were not significantly different, RAD6B expression levels were noticeably more heterogeneous across the melanoma cases. Using an F-test, we analyzed the equality of variances between RAD6A and RAD6B expression levels. The RAD6B expression level was 1.47-fold more heterogeneous across the samples as compared to RAD6A (r = 1.472; 95% CI, 1.229 to 1.764; *p* < 0.001; Figure 1A). Similarly, the copy number variations of the *RAD6B* gene was 5.65 times more heterogeneous as compared to that of *RAD6A* (r = 5.664; 95% CI, 4.727 to 6.786; *p* < 0.001; Figure 1A).

### 3.2. Melanoma Cell Lines and Patient-Derived Metastatic Melanomas show RAD6B mRNA Alterations Resulting from Alternative Splicing 

Since the TCGA data in Figure 1A revealed significant heterogeneity in RAD6B expression levels and copy number alterations as compared to RAD6A, we performed RT-PCR analysis of RAD6B transcripts amplified from normal melanocytes (HEMa-LP), melanoma lines (A375, A2058, M14), and patient-derived melanoma brain metastasis (Mel-Met-14-089 and Mel-Met-14-108) using RAD6B specific primers that span the entire RAD6B coding region.

**Figure 2:**
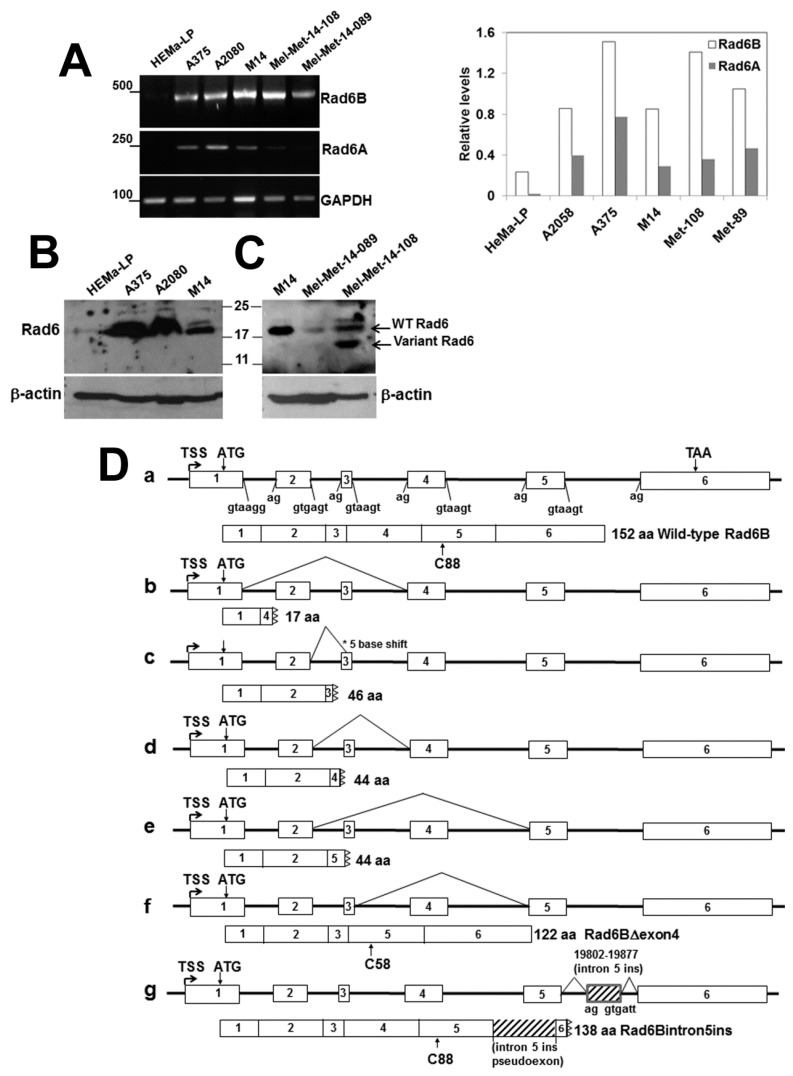
**RAD6B is robustly expressed in melanoma lines and shows abnormal processing of transcript splicing.** (**A**) RT-PCR analysis of RAD6A and RAD6B in normal (HeMa-LP) melanocytes, melanoma cell lines and clinical melanoma brain metastasis samples Mel-Met-14-108 and Mel-Met-14-089. RAD6A and RAD6B levels normalized to GAPDH are shown in the graph on the right. (**B**) and (**C**), Western blot analysis of RAD6 in HeMa-LP, melanoma lines and clinical melanomas. Note that since the RAD6 antibody does not distinguish RAD6A and RAD6B, the protein is indicated as RAD6. The presence of a 14 kDa variant RAD6 protein in clinical melanoma is indicated. (**D**) (**a**) Schematic structure of the human *RAD6B* gene and splice junctions. The wild-type protein with the active cysteine (C)88 is indicated below. (**b**–**g**) Schematic mutations of RAD6B splicing mutations identified in melanoma lines and clinical melanoma brain metastases. The 122 amino acid RAD6BΔexon4 mutant (**f**) was identified in M14 and A2058 melanoma lines, and the 138 amino acid RAD6Bintron5ins (**g**) splice mutant was identified in the M14 line. The positions of the active cysteine residue in the splice mutants are shown.

Whereas all melanoma samples robustly expressed RAD6B, expression of RAD6B was weak in HEMa-LP cells (Figure 2A). RT-PCR analysis of RAD6A showed that its expression was ~2–3-fold lower than RAD6B in all melanoma lines and was negligible in HeMa-LP cells (Figure 2A and graph). The RT-PCR data were corroborated by Western blot analysis, which showed strong RAD6 expression in the melanoma lines and very weak expression in HEMa-LP cells (Figure 2B,C). The RAD6 antibody reactive to the carboxyl terminus detects wild-type RAD6 and only variants of RAD6 containing the carboxyl terminus as in Mel-Met-14-108 (Figure 2C). Furthermore, since RAD6A and RAD6B share ~95% amino acid sequence identity, our RAD6 antibody does not distinguish the two paralogs. Thus, the endogenous proteins detected in Western blots are labeled as RAD6 rather than RAD6A or RAD6B. However, since our RT-PCR analysis showed RAD6B expression to be stronger than RAD6A, we surmise that the RAD6 protein detected in normal melanocytes and melanomas is probably derived predominantly from the RAD6B gene, as is the case in breast cancer [25,26].

The *RAD6B* gene is 24,929 bp in length (Accession No. DQ090910) and encompasses six relatively small exons that are 464, 81, 26, 90, 89, and 1884 bp, separated by introns of 2743, 2205, 4025, 7518 and 1811 bp, respectively, and it transcribes a full-length mRNA of 2634 bases (Accession No. NM_003337) (Figure 2Da). The wild-type RAD6B protein is 152 amino acids long with a molecular weight of ~17 kDa, and the cysteine (Cys88) residue critical for its E2 ubiquitin conjugating activity is located within exon 5 (Figure 2Da). To determine the sequence integrity of the RAD6B transcripts amplified in Figure 2A, the RT-PCR products from HEMa-LP and melanomas were gel-purified, subcloned into the PCR2.1-Topo vector, screened by restriction digestion, and clones selected from each cell line were subjected to sequence analysis. Wild-type RAD6B was the predominant transcript based upon initial screening analysis. Analysis of the HEMa-LP cells did not identify clones showing variation(s) in size compared to the wild-type RAD6B by restriction analysis, and sequencing of four recombinant clones that yielded transcripts matching the wild-type RAD6B transcript revealed only wild-type sequences. Sequenced reads similarly confirmed that all melanoma cell lines including patient-derived metastatic melanomas express the wild-type RAD6B transcript. Besides the wild-type transcript, we identified several alternatively spliced forms of RAD6B transcripts in the melanoma lines and clinical melanomas (Figure 2Db–g). Sequence analysis revealed spliced variants with exon skipping events, particularly those involving exons 2, 3 and/or 4 (Figure 2D (b,d,e,f)). Apart from exon skipping, we also found one instance of an alternate 3′ splice site selection in A2058 cells and clinical melanomas, which resulted in the loss of five bases from the start of exon 3 (Figure 2Dc). Many of these splice variants, with the exception of exon 4 deletion variant (RAD6BΔexon4) in Figure 2Df, translated to premature termination codons due to frameshifts producing truncated RAD6B (Figure 2Db–e). Interestingly, these alternate splicing events excluded exon 5 as it was correctly retained in all the transcripts analyzed (Figure 2Db–g). Our analysis identified the RAD6BΔexon4 variant in M14 and A2058 cells, which resulted from exon 4 skipping (deletion of amino acids 51–80), resulting in an in-frame deletion that translates into a 122 amino acid mutant RAD6B protein with an intact catalytic region and a predicted molecular mass of 14 kDa (Figure 2Df). This RAD6BΔexon4 variant has been previously identified as RAD6B isoform 2 (Accession #P49459-2). Another previously identified splice variant RAD6B isoform 6 (Accession #PN149972.1) was identified only in M14 cells and resulted from the utilization of viable alternate 5′ and 3′ splice sites within intron 5. This resulted in the inclusion of a 76 base pseudo exon into the RAD6B mRNA (Figure 2Dg). Although the insertion of this pseudo exon did not impact downstream splicing with exon 6, it however led to premature termination due to a frameshift mutation (Figure 2Dg).

Translation of this RAD6Bintron5ins splice variant is predicted to produce a 138 amino acid catalytically active mutant RAD6B protein lacking exon 6 but containing an in-frame intron 5-derived 25 amino acid peptide at the C-terminus. Although transcripts for the RAD6BΔexon4 and RAD6Bintron5ins splice variants were not detectable in Mel-Met-14-108 and Mel-Met-14-089 patient-derived melanomas, besides the wild-type RAD6 protein, a 14 kDa RAD6 band corresponding to the size of the translated RAD6BΔexon4 transcript (based on its amino acid sequence) was detected by Western blotting in Mel-Met-14-108 patient samples (Figure 2C). The RAD6BΔexon4 variant transcript was expressed in M14 and A2058 cells; however, we did not detect its translated product in these cells by Western blotting. It is possible that the wild-type RAD6B protein abundance masks the detection of the splice mutants or that the steady-state levels of the mutant proteins may be low because of decreased stability.

### 3.3. RAD6BΔExon4 and RAD6Bintron5ins Splice Variants are Functional

**Figure 3:**
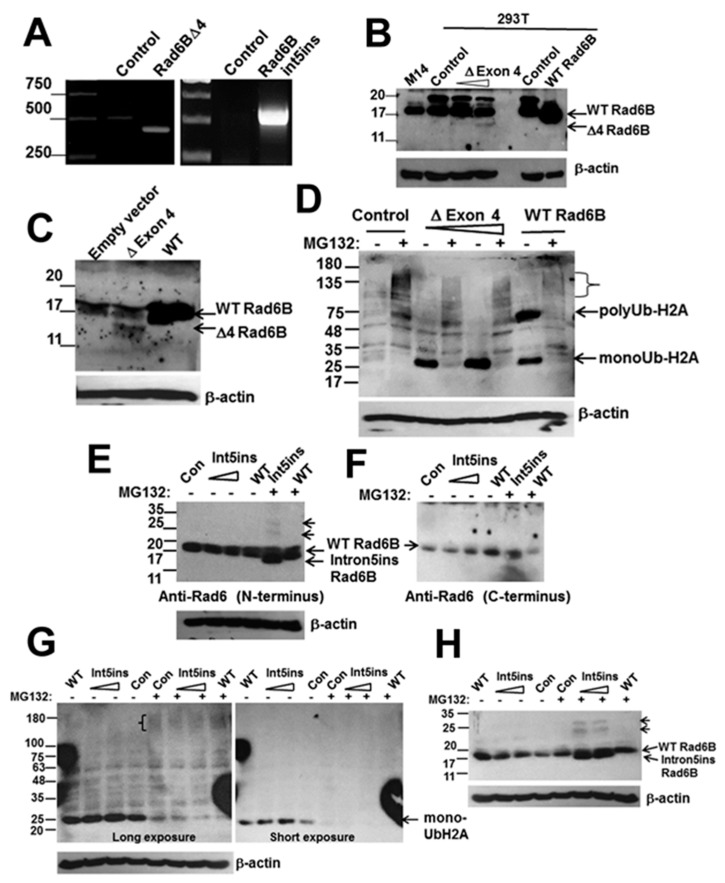
**The splice mutants RAD6BΔexon4 and RAD6Bintron5ins are catalytically active.** (**A**) RT-PCR analysis of RAD6BΔexon4 and RAD6Bintron5ins expressions in 293T or COS7 transfected cells. (**B**) and (**C**), Western blot analysis of RAD6B using the C-terminus reactive antibody in control, wild-type (WT) RAD6B and RAD6BΔexon4 transfected 293T (**B**) and COS7 (**C**) cells. In (**B**), M14 cells were used as a melanoma control for biological validation of endogenous and exogenous WT RAD6B expressed in 293T cells. (**D**) In vivo ubiquitination activities of WT RAD6B and RAD6BΔexon4 were assessed in transfected COS7 cells with and without MG132 treatment by Western blotting with K119 ubiquityl histone H2A antibody. The positions of the mono- and polyubiquitinated H2A are indicated. The high molecular weight species of ubiquitinated histone H2A accumulating in MG132 treated control, RAD6BΔexon4 or WT RAD6B transfected cells are denoted by a bracket. (**E**) Western blot analysis of RAD6B using the N-terminus reactive antibody in control, WT RAD6B and RAD6B intron5ins transfected COS7 cells with and without MG132 treatment. Note that the 15 kDa intron5ins mutant is detected only in MG132 treated cells. Also note the presence of higher molecular weight RAD6B mutant bands in MG132 treated cells indicated by short arrows. (**F**) Re-probing of the stripped blot in (**E**) with RAD6 antibody specific for the C-terminus. Note that the nascent and higher molecular weight forms of RAD6Bintron5ins are not detected with the C-terminus reactive RAD6 antibody. (**G**) In vivo ubiquitination activity of RAD6Bintron5ins mutant in COS7 cells with and without MG132 treatment. High molecular weight or polyubiquitinated histone H2A detected in MG132 treated cells is indicted by a bracket. (**H**) The blot in (**G**) was stripped and re-probed with RAD6 antibody specific for the N-terminus and β-actin. Note the commensurate appearance of the RAD6B mutant and its mono- and di-ubiquitinated forms in MG132 treated cells.

To determine whether RAD6BΔexon4 and RAD6Bintron5ins variants are indeed translated, we inserted them into pCI-neo expression vector under the control of the constitutive CMV promoter. RAD6BΔexon4, RAD6Bintron5ins, and WT RAD6B vectors were transiently transfected into 293T or COS7 cells, and 48 h later they were processed for RNA and protein analysis. RT-PCR analysis confirmed the capability and size of the RAD6BΔexon4-derived 423 base transcript in transfected cells as compared to the 513 base endogenous wild-type RAD6B mRNA in untransfected or control 293T or COS7 cells (Figure 3A). Western blot analysis with the RAD6 antibody reactive to the carboxyl terminus showed expression of a 14 kDa protein in both 293T (Figure 3B) and COS7 (Figure 3C) cells confirming translation of the RAD6BΔexon4 transcript. However, despite robust mRNA expression, the steady-state levels of RAD6BΔexon4 variant protein were >50-fold lower than those derived by ectopic expression of WT RAD6B vector transfected at ~5-fold lower levels. These data suggest that the RAD6B protein lacking exon 4 may be less stable as compared to the WT RAD6B protein. Treatment with MG132 increased the steady-state levels of native RAD6BΔexon4 and revealed the presence of high molecular weight forms, potentially representing polyubiquitinated RAD6BΔexon4 (Supplementary Figure S1A). Next, to assess the functional activity of the deletion mutant, COS7 lysates prepared from WT RAD6B or RAD6BΔexon4 variant transfected cells with and without MG132 treatment were subjected to Western blot analysis with ubiquityl-histone H2A antibody, as RAD6B is a major ubiquitinator of histone H2A [28]. In the absence of MG132 treatment, a dose-dependent increase in monoubiquitinated histone H2A was seen in RAD6BΔexon4 transfected cells as compared to control untransfected cells (Figure 3D). WT RAD6B transfected cells showed more robust histone H2A ubiquitination as bands corresponding to both mono- and polyubiquitinated histone H2A were detected (Figure 3D). Treatment with MG132 proteasome inhibitor induced the accumulation of high molecular weight polyubiquitinated histone H2A (indicated by brackets in Figure 3D), which was accompanied by concomitant decreases in monoubiquitinated histone H2A in both RAD6BΔexon4 and WT RAD6B expressing cells (Figure 3D). The polyubiquitinated chains apparently masked the K119 monoubiquitinated epitope recognized by the antibody, as the intensities of the higher molecular weight species were considerably weaker than those with one or few ubiquitin moieties (Figure 3D). These data confirm the functional activity of RAD6BΔexon4 variant and suggest that the peptide sequence encompassing exon 4 is dispensable for its catalytic activity but may be required for bolstering the catalytic activity via stabilization.

RT-PCR analysis of RAD6Bintron5ins variant transfected COS7 cells confirmed the expression of the RAD6Bintron5ins-derived 589 base transcript (Figure 3A). Since the RAD6Bintron5ins variant lacks the carboxyl terminus epitope recognized by our RAD6 antibody [25], western blot analysis was performed with a RAD6 antibody reactive to its amino terminus. A 15 kDa (138 amino acid) RAD6Bintron5ins-derived product was detected in MG132 treated cells, and either weakly (Supplementary Figure S1A) or not in the corresponding untreated (Figure 3E) cells. Anti-RAD6 reactive ~23 and 31 kDa bands potentially representing the mono- and di-ubiquitinated forms of RAD6Bintron5ins variant were also detected in MG132 treated cells, suggesting that this variant may be susceptible to 26S proteasomal degradation (Figure 3E). Treatment with MG132 also enhanced the levels of nonubiquitinated native RAD6Bintron5ins protein (Figure 3E,H and Figure S1A). Since the disassembly of polyubiquitin chains is also blocked when the catalytic activity of the proteasome is inhibited by MG132, this results in depletion of the free ubiquitin pool in the cells [29], and by doing so the RAD6B variants are not ubiquitinated, leading to their stabilization. Unequivocal confirmation of RAD6Bintron5ins protein ubiquitination could not be made as an N-terminus immunoreactive RAD6B antibody that is suitable for immunoprecipitation is currently not available. Since the RAD6Bintron5ins variant lacks the carboxyl terminus epitope of our RAD6 antibody, we stripped and re-probed the blot with carboxyl terminus immunoreactive RAD6 antibody to verify the integrity of the expressed band. While endogenous and exogenous WT RAD6B were detected, the RAD6Bintron5ins variant band and the putative ubiquitinated forms were not observed validating the spliced mutant (Figure 3F). Western blot analysis with anti-ubiquityl-histone H2A antibody was performed to assess the catalytic activity of RAD6Bintron5ins variants. A dose-dependent increase in monoubiquitinated histone H2A was seen in cells transfected with 3 or 6 μg of RAD6Bintron5ins variant as compared to control cells (Figure 3G, short and long exposures). H2A ubiquitination in WT RAD6B transfected cells was not as robust as in Figure 3D, as cells were transfected with only 1 μg of the construct. Similar to the data in Figure 3D, MG132 treatment resulted in loss of the monoubiquitinated histone H2A band and the appearance of high molecular weight polyubiquitinated histone H2A species (Figure 3G, bracket). Re-probing the stripped blot with the amino terminus reactive RAD6 antibody again revealed the RAD6Bintron5ins mutant and its mono- and di-ubiquitinated species only in MG132 treated cells, confirming its susceptibility to proteasomal degradation (Figure 3H). These data confirm the expression and ubiquitin conjugating activity of the RAD6Bintron5ins variant protein.

### 3.4. Homology/Template-Based Modeling of RAD6BΔexon4 and RAD6Bintron5ins 3D Structures

**Figure 4:**
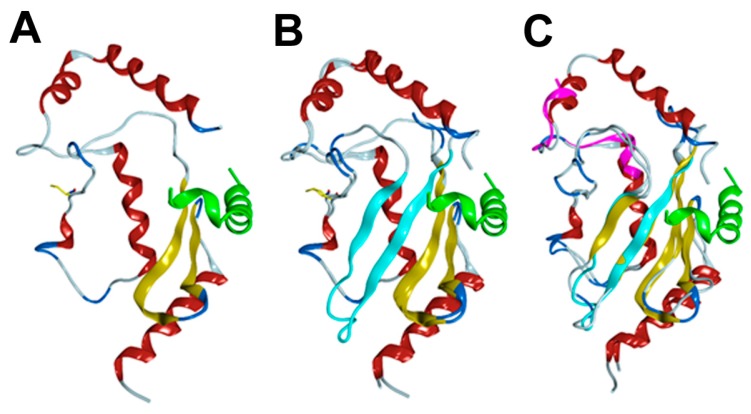
**Homology models of RAD6BΔexon4 and RAD6Bintron5ins.** (**A**) Model of RAD6BΔexon4. The catalytic cysteine residue is shown as a yellow line. RAD18 is shown in green. (**B**) Overlay of RAD6BΔexon4 and wild-type RAD6B from PDB file 2YBF. Beta sheets 3 and 4 of the wild-type RAD6B are shown in light blue. RAD18 is shown in green. (**C**) Overlay of RAD6Bintron5ins with wild-type RAD6B from PDB file 2YBF. The insertion is shown in magenta.

The 3D structures of RAD6BΔexon4 and RAD6Bintron5ins variants were created by homology/template modeling using the PDB file 2YB6 for RAD6. In both cases, 50 templates were generated. For both proteins, the top five models were ubiquitin conjugating enzymes with sequence identities ranging from 96% to 100% for RAD6BΔexon4 and from 84% to 88% for RAD6Bintron5ins. The Global Model Quality Estimation (GMQE) was 0.98 for RAD6BΔexon4 and 0.85 for RAD6Bintron5ins, indicating that both models are predicted to be highly reliable. The deletion of exon 4 in RAD6B removes amino acids 51–80 (Figure 4A), which eliminates beta-sheets 3 and 4 from the wild-type structure (Figure 4B). The connection of D50 to V81 results in a linker that connects loops on either side of the beta-turn-beta motif that is eliminated from RAD6B (Figure 4A,B). This connection, however, does not disrupt the tertiary structure of the protein or the catalytic cysteine residue, which is consistent with the results in Figure 3D showing intact ubiquitin conjugating activity for RAD6BΔexon4.

The binding site for RAD18, the cognate E3 ligase for RAD6, located at the carboxyl terminus of RAD6B is intact [30], although potential interactions at the C-terminus of RAD18 may be disrupted due to the absence of beta-sheets 3–4. Beta-sheet 2 forms an extensive series of hydrogen bonds with beta-sheet 3 in the wild-type RAD6B structure (Figure 4B). These interactions are absent in RAD6BΔexon4 as this disruption removes a series of hydrogen bonds that connect beta-sheets 1 and 2, which are critical for RAD18 binding to the loop that holds the catalytic cysteine residue (Figure 4A,B). Thus, while the overall structure of RAD6BΔexon4 is very similar to wild-type RAD6B, it is likely that the deletion of the two beta-sheets would affect the dynamics and stability of the protein, and possibly the response to the binding to RAD18 protein.

The intron 5 insertion replaces amino acids 111–151 from the wild-type RAD6B protein with CLALLPRLECNGTILAHRNLCLPGSISAG. A homology model was created, which ended at R128 with the last C-terminal 11 residues not modeled (Figure 4C). Numerous attempts to find sequence similarity to experimentally derived structures failed, and thus a model containing the entire sequence of the intron 5 insertion could not be generated. The model lacked alpha-helix 5 and part of helix 4. The C-terminus of RAD6B interacts with RAD18 and forms part of the binding site for RAD18. If we assume that the insertion follows the same geometry of helices 4 and 5, the insertion would shorten the RAD6B isoform by 14 amino acids, which could alter the binding affinity for RAD18. There are no changes observed for the catalytic cysteine residue, although there are changes to the sequence of amino acids around the cysteine. Our experimental data in Figure 3G indicate that this does not affect its catalytic activity. Insertion of the intron 5-derived pseudo exon introduces three new cysteine residues, and our data suggest that these cysteine residues could potentially act as ubiquitin acceptor sites and partake in RAD6B variant autoubiquitination as mono- and di-ubiquitinated forms of the variant were detected in MG132 treated cells (Figure 3E,H).

### 3.5. RAD6B Variant Expression is a Hallmark of Clinical Melanomas

**Figure 5:**
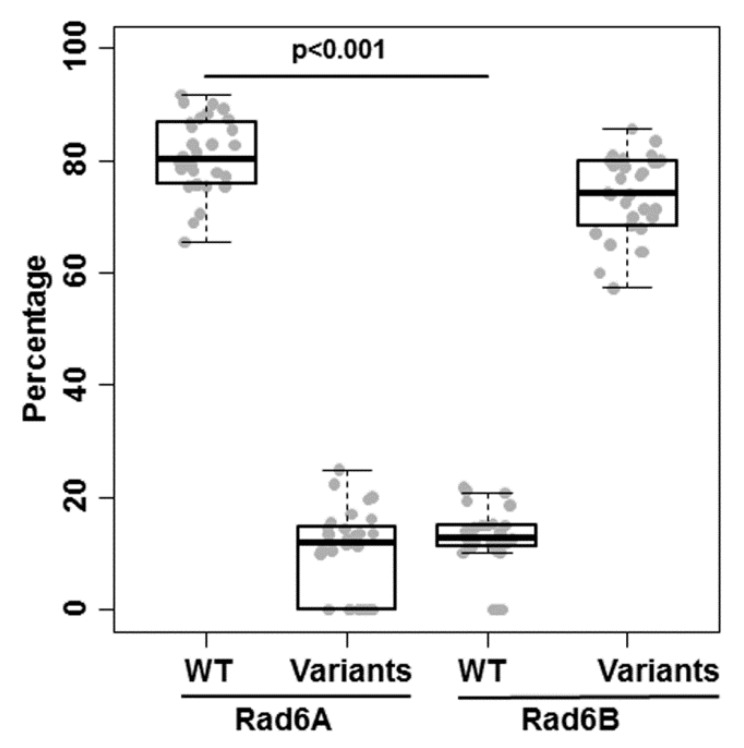
**Whole exome sequence analysis of RAD6A and RAD6B transcripts in primary and metastatic cutaneous melanoma xenografts.** The percentages of wild-type (WT) and variant forms of RAD6A and RAD6B transcripts identified in 30 melanoma patients are shown. Comparisons of the percentages of WT RAD6A and WT RAD6B were made using the Wilcoxon test for paired samples. The box plots represent the minimum, Q1, median, Q2, and maximum of percentages. The details of RAD6A and RAD6B variants, patient demographics and stage of disease are provided in Supplementary Tables S1 and S2.

To determine whether RAD6B variant expressions reflect a general property of melanomas, we performed variant analysis of human RAD6A and RAD6B transcripts uncovered by WES analysis of 30 patient-derived melanoma xenografts [24]. The percentages of wild-type RAD6A and wild-type RAD6B relative to their corresponding variants were compared using the Wilcoxon test for paired samples. Significant differences in the average numbers of variants were found for RAD6A versus RAD6B (*p* < 0.001; Figure 5). Whereas the majority of RAD6A transcripts (range 69–91%) coded for the 152 amino acid wild-type protein (ENST00000371558) with a small proportion coding for a 149 amino acid RAD6A protein (ENST00000346330), <20% (range 10.3–18.6%) of the RAD6B transcripts coded for the 152 amino acid wild-type protein (ENST00000265339) (Figure 5 and Supplementary Tables S1 and S2). All melanomas co-expressed varying proportions of nonsense, 138 amino acid intron 5 insertion variant (ENST00000506787), and/or 141 (ENST00000507277) amino acid RAD6B isoforms that contain the catalytic domain (Supplementary Table S2). RAD6B variants were co-expressed in 30/30 clinical melanomas analyzed and represent the majority of the RAD6B transcriptome, with the intron 5 insertion variant ENST00000506787 expressed in >50% of patient-derived melanomas. Furthermore, since many of the RAD6B variants are common across melanomas, regardless of stage and gender (Supplementary Table S2), our data suggest that the expression of these precisely spliced functional RAD6B isoforms could contribute to melanoma pathogenesis and account for the RAD6B molecular heterogeneity in melanoma patients.

## 4. Discussion

We have previously shown that RAD6 is an early marker of cutaneous melanoma development, as increases in its expression correlate with increases in melanocytic proliferation and the disruption of the normal melanocyte to keratinocyte ratio in the skin [15]. RAD6 is weakly expressed in nevi but is overexpressed in primary and metastatic melanomas, implicating an important role for RAD6 in melanoma development, progression, and metastasis [16]. In this study, we have identified for the first time several co-expressed RAD6B splice variants that represent truncated RAD6B or modified functional versions of the parent RAD6B in melanoma cell lines and in patient-derived melanoma brain metastases but not in normal melanocytes. TCGA analysis showed that gene expression and copy number variations are significantly more heterogeneous for *RAD6B* than *RAD6A* in melanomas. These data are corroborated by WES analysis of patient-derived primary and metastatic melanoma xenografts, which not only showed co-expression of RAD6B variants with wild-type RAD6B but also showed that these variants constitute the majority of the RAD6B transcriptome in contrast to RAD6A, which was predominantly represented by the wild-type form. Genomic analysis carried out in tumors and their matched patient-derived xenografts showed a tight correlation, as 88% of the mutations identified in the parental tumors were found to be present in the corresponding xenografts, establishing the validity of the WES data [24]. The identification of various co-expressed RAD6B splice variants in clinical melanomas provides a mechanistic basis for RAD6B molecular heterogeneity.

Alternative splicing of pre-mRNA allows a gene to encode many more products, thus increasing the heterogeneity of the transcript and potential modification of the wild-type protein. Our data show that the major mechanism of RAD6B splice variant generation utilized in melanoma involves exon skipping that is localized to exons 2, 3 or 4. Interestingly, splice variants involving exons 5 and 6 were not observed in any of the melanoma samples analyzed. The fact that the RAD6B alternative transcripts identified in melanoma cells are not unique to melanomas suggests that these variants result from nontrivial splicing events, some of which are nonfunctional at the mRNA or protein level, resulting in nonsense mediated mRNA decay, a translation-coupled mechanism that eliminates transcripts containing premature translation termination codons. In mammalian cells, nonsense mediated decay has also been linked to pre-mRNA splicing [31]. The RAD6BΔexon4 variant identified in M14 and A2058 melanoma lines has been described previously [32]. We have also identified a previously reported 138 amino acid RAD6B mutant (ENST00000506787.5; HOYA80-1) resulting from a nontrivial splicing event within intron 5, and we have demonstrated for the first time that this variant containing the intron 5-derived pseudo exon is catalytically active. The relevance of this variant in melanoma is exemplified by our WES data, which showed its presence in 50% of the patient-derived primary and metastatic melanomas. Interestingly, this splice mutant annotated as RAD6B isoform 6 has been found in other organisms including chimpanzees (Accession # PNI49972), indicating its evolutionary conservation. Considering the preponderance of RAD6B splice variants with exon deletions in melanoma lines, the failure to detect these isoforms in patient-derived melanoma xenografts potentially reflects the limitations of WES analysis.

Spliceosomal errors resulting in the production of aberrantly spliced transcripts are rare in normal cells, whereas they appear to be an intrinsic property of tumor cells [33]. Alterations in splice site usage or selection have been shown to affect genes implicated in cancer susceptibility (BRCA1) and in tumor development or progression (e.g., MDM2, CD44) [34,35,36]. Consistent with these data, RAD6B splice variant expressions appear to be melanoma-specific as they were not seen in normal melanocytes. Co-expression of variant forms may reflect RAD6B transcription rate, as melanomas robustly expressing wild-type RAD6B also show co-expression of variant forms whereas normal melanocytes that show weak RAD6B expression do not display splice variant expressions. We posit that the robust transcription/elongation rates of wild-type RAD6B in melanomas could influence alternative splicing choices and compromise the splicing fidelity. The co-expressed splice variants could positively or negatively influence the parent RAD6B transcription, catalytic activity, interactions with other proteins, or introduce new protein–protein interactions as in the intron 5 insertion splice variant. Based on the localization of exon 4 (51–80 amino acids) peptide in the RAD6B 3D model, loss of the 51–80 amino acid β-strand in the RAD6BΔexon4 variant is predicted to not impact the structural features required for its ubiquitin conjugating function as well as the carboxyl end binding site (amino acids 141–149) required for interactions with RAD18 that is necessary for replicative bypass or translesion synthesis [30]. Consistent with the modeling data, we show that the RAD6BΔexon4 variant is indeed expressed as a 14 kDa protein, and the RAD6Bintron5ins variant is expressed as a 15 kDa protein. Our data from in vivo ubiquitination assays confirm that both RAD6BΔexon4 and RAD6Bintron5ins variants are biologically active as they are able to catalyze the monoubiquitination of histone H2A in vivo. Since efficient histone H2A ubiquitination occurs despite the presence of low or negligible levels of the variant proteins in the absence of MG132, our data suggest that once expressed these variant proteins are quickly able to catalyze histone H2A monoubiquitination, and since monoubiquitinated histone H2A is stable this results in elevated steady-state levels. In contrast, these variant proteins have poor stability because of proteasomal degradation, and consequently fail to accumulate sufficient steady-state levels required for detection by the Western Blotting conditions employed. This premise is supported by MG132 treatment-induced accumulation of both nonubiquitinated (native) RAD6BΔexon4 and RAD6Bintron5ins proteins as well as higher molecular weight or polyubiquitinated forms of these proteins. Since MG132 treatment results in similar polyubiquitination of histone H2A by RAD6BΔexon4 and RAD6Bintron5ins as WT RAD6B, our data suggest that the low stabilities of variant RAD6B rather than their specific activities contribute to their decreased robustness as compared to WT RAD6B. It is of interest to note that the inclusion of three additional cysteine residues from the intron 5-derived pseudo exon could potentially contribute to RAD6B mutant autoubiquitination and susceptibility to proteasomal degradation. WT RAD6B did not show similar autoubiquitination and stability changes (Supplementary Figure S1B), further supporting the impact of the extra cysteines in RAD6B autoubiquitination. A potential mechanism of RAD6Bintron5ins autoubiquitination may involve the transfer of the activated ubiquitin from active Cys88 to one or more of the three extra cysteine residues in the variant by trans-thiolation and subsequent transfer of the ubiquitin moieties to lysine residues in RAD6B. In such a case, the RAD6Bintron5ins variant would possess not only E2 ubiquitin conjugating activity but also E3 ubiquitin ligase activity, which could obviate the need for coordination with its cognate E3 ligases. In this regard, it is interesting to note that this variant is predicted to lose the RAD18 interaction surface at the carboxyl terminus and potentially its postreplicative bypass function. The presence of an intron 5-derived pseudo exon has been identified in UBE2B isoform 6 (Accession # PN149972.1), and bears ~86% homology to amino acids 237–257 of human ubiquitin carboxyl terminus hydrolase 4 (USP4) isoform C (Accession # NP_0012388061.1), and 82–91% homology to various isoforms including β1,3 N-acetyl galactosaminyl transferase 2 isoform X3 (Accession # XP_010382964). Since this intron 5-derived sequence has been observed only in isoforms, this suggests conservation of this intron sequence in other genes and similar utilization of this sequence by alternative splicing.

RAD6B knockout male mice are sterile [37], indicating the essential role of RAD6B in spermatogenesis [38]. Consistent with its importance in sperm development, a diverse array of RAD6B splice variants were observed in oligozoospermic men as compared to normozoospermic men, suggesting that aberrant RAD6B mRNA processing may be an important player in low sperm counts [32]. Interestingly, some of the splice variants identified in melanoma are identical to those observed in the oligozoospermia samples. As in melanoma cells, exon skipping events involving exons 2, 3 and/or 4 were the most common alternative splicing events observed in oligozoospermic men [32]. However, in oligozoospermic men, exon skipping or altered 3′ or 5′ splicing also impacted the catalytic domain containing exon 5, which was unaffected in melanoma cells. 

Mutations in splice sites, polypyrimidine tract, branch point, and intronic or exonic enhancers and silencers can affect splicing [39]. Our data suggest that somatic mutations in exons or splice sites cannot explain the recurrent RAD6B isoform switching patterns, as similar alternative splicing events were observed across multiple melanoma cell lines analyzed. Consistent with this, sequence analysis of the flanking intronic regions for all coding exons of the RAD6B gene in oligozoospermia samples have failed to identify splice site mutations [32]. The length of the upstream introns or inefficiency of 5′ splice sites have been proposed to explain exon skipping [39,40]. However, our data suggest that intron lengths may not be a major factor for exon skipping, as introns flanking the skipped exons 2, 3 or 4 range from 2.7 to 4 kb, whereas the intron upstream of exon 5 that was rarely skipped was the longest, at ~7.6 kb. We also rule out the contribution of different splice site strengths for splicing errors, as melanoma cells display a diverse array of exon skipped transcripts: Δ2, Δ3, Δ4, Δ2/Δ3, and Δ3/Δ4. Mutation, misexpression or mislocalization of RNA binding proteins SRSF1, hnRNPs, or RBM regulating splicing have been reported in several cancers [39]. In this regard, a recurrent mutation at R625 of SF3B1, a spliceosomal protein, has been reported in mucosal and anorectal melanomas [41,42], and patient specimens with SF3B1 mutation showed higher incidences of mRNA splice variants for ABCC5, CRNDE, RPL31 and TEME14C genes [42].

We have identified recurrent RAD6B isoforms in melanoma samples having at least two of them with intact catalytic function. Since these splice variants were not detected in normal melanocytes, we posit that mis-splicing may be a consequence of altered events during tumorigenesis and that the variant isoforms may directly contribute to melanoma development and/or progression. It is also possible that the splice variants are not detected in normal melanocytes because of the weak transcriptions of wild-type RAD6B, as high transcription rates have been reported to affect splicing fidelity [43]. Since our commonly used RAD6 antibody recognizes an epitope in the carboxyl terminus, this has limited detection of truncated RAD6B forms as well as RAD6B with a modified carboxyl end, such as the intron 5 insertion variant that is present in >50% of the clinical melanomas analyzed. Using the amino terminus-reactive RAD6 antibody has permitted the detection of the RAD6Bintron5ins protein, and our data show that some of these functionally active variants fail to accumulate sufficient steady-state levels due to their susceptibility to proteasomal degradation. We posit that the production of pathogenic isoforms from nontrivial splicing events, such as inclusion of the intron 5-derived pseudo exon in the RAD6Bintron5ins variant, could create binding sites for new interaction partners or generate novel epitopes that could be exploited as targets for therapy.

## Supplementary Materials

The following are available online at https://www.mdpi.com/2073-4409/8/11/1375/s1, Figure S1: Analysis of MG132 effects on wild-type (WT), RAD6Bintron5ins, and RAD6BΔexon4 protein levels. Table S1: Whole exome sequence analysis of RAD6A (UBE2A) transcripts in patient-derived melanoma xenografts. Table S2: Whole exome sequence analysis of RAD6B (UBE2B) transcripts in patient-derived melanoma xenografts.
