# Supplementary material for: Alternative Splicing of RAD6B and Not RAD6A Is Selectively Increased in Melanoma: Identification and Functional Characterization
Source: Cells. 2019 Nov 1;8(11):1375. doi: 10.3390/cells8111375 (PMC6912459; doi:10.3390/cells8111375)
Supplement: Supplementary file 1 [file cells-08-01375-s001.pdf]

SUPPORTING INFORMATION

Table 1. Whole exome sequence analysis of Rad6A (UBE2A) transcripts in patient-derived melanoma xenografts. Data provided by Champions Oncology, Inc.

Number	Tumor	Stage	Sex	Race	Age	Gene	Transcript ID
1	Primary	IV	Male	NA	56	UBE2A	ENST00000371558: 82.83% ENST00000371569: 10.55%
2	Metastatic	IV	Male	Caucasian	89	UBE2A	ENST00000371558: 75.45% ENST00000346330: 13.56%
3	Metastatic	III	Male	Caucasian	73	UBE2A	ENST00000371558: 75.8% ENST00000346330: 22.48%
4	NA	NA	NA	NA	NA	UBE2A	ENST00000371558: 87.67%
5	Metastatic	III	Female	Caucasian	73	UBE2A	ENST00000371558: 87.43%
6	Metastatic	IV	Male	NA	71	UBE2A	ENST00000371558: 75.46% ENST00000371569: 13.51%
7	Metastatic	IV	Male	NA	56	UBE2A	ENST00000371558: 78.58% ENST00000346330: 13.34%
8	Metastatic	III	Male	Caucasian	64	UBE2A	ENST00000371558: 78.16%
9	Primary	II	Male	Caucasian	84	UBE2A	ENST00000371558: 81.5% ENST00000346330: 15.64%
10	Metastatic	IV	Male	NA	51	UBE2A	ENST00000371558: 80.8%
11	Metastatic	IV	Male	NA	74	UBE2A	ENST00000371558: 70.51% ENST00000371569: 13.62% ENST00000346330: 11.33%
12	Metastatic	IV	Male	NA	53	UBE2A	ENST00000371558: 65.49% ENST00000469205: 17.08%
13	Metastatic	II	Male	NA	50	UBE2A	ENST00000371558: 90.44%
14	Metastatic	III	Male	Caucasian	63	UBE2A	ENST00000371558: 85.61% ENST00000371569: 10.04%
15	NA	NA	NA	NA	NA	UBE2A	ENST00000371558: 79.81% ENST00000346330: 14.49%
16	Primary	IV	Female	NA	61	UBE2A	ENST00000371558: 82.92% ENST00000346330: 11.5%
17	Primary	I	Male	Caucasian	55	UBE2A	ENST00000371558: 79.3% ENST00000371569: 10.6%
18	Metastatic	IV	Female	Caucasian	35	UBE2A	ENST00000371558: 88.46%
19	Metastatic	IV	Male	Caucasian	43	UBE2A	ENST00000371558: 86.81% ENST00000346330: 11.99%
20	Primary	III	Male	Caucasian	40	UBE2A	ENST00000371558: 91.78%
21	Metastatic	III	Male	Caucasian	76	UBE2A	ENST00000371558: 85.96%
22	Metastatic	IV	Male	Caucasian	87	UBE2A	ENST00000371558: 89.24%
23	Metastatic	IV	Male	Caucasian	68	UBE2A	ENST00000371558: 77.26% ENST00000371569: 13.58%
24	Primary	II	Male	NA	74	UBE2A	ENST00000371558: 75.81% ENST00000371569: 16.22%
25	Primary	IV	Male	NA	56	UBE2A	ENST00000371558: 90.25%
26	Metastatic	II	Male	Caucasian	39	UBE2A	ENST00000371558: 79.14% ENST00000346330: 19.62%
27	Primary	II	Male	Caucasian	87	UBE2A	ENST00000371558: 75.52% ENST00000371569: 11.72%
28	Local Metast	IV	Female	Caucasian	56	UBE2A	ENST00000371558: 77.96% ENST00000346330: 14.63%
29	Primary	II	Male	Caucasian	49	UBE2A	ENST00000371558: 83.05% ENST00000371569: 12.58%
30	Primary	IV	Male	Caucasian	41	UBE2A	ENST00000371558: 68.98% ENST00000371569: 20.16%

ENST00000371558, wild-type Rad6A (UBE2A)

Table 2. Whole exome sequence analysis of Rad6B (UBE2B) transcripts in patient-derived melanoma xenografts. Data provided by Champions Oncology, Inc.

Number	Tumor	Stage	Sex	Race	Age	Gene	Transcript ID
1	Primary	IV	Male	NA	56	UBE2B	ENST00000499038: 66.2% ENST00000506787: 14.85%
2	Metastatic	IV	Male	Caucasian	89	UBE2B	ENST00000499038: 49.58% ENST00000507277: 20.44% ENST00000265339: 11.17%
3	Metastatic	III	Male	Caucasian	73	UBE2B	ENST00000507277: 49.97% ENST00000506787: 13.82% ENST00000265339: 11.26%
4	NA	NA	NA	NA	NA	UBE2B	ENST00000499038: 49.16% ENST00000507277: 24.9% ENST00000265339: 14.04%
5	Metastatic	III	Female	Caucasian	73	UBE2B	ENST00000507277: 53.8% ENST00000506787: 20.5% ENST00000265339: 15.09%
6	Metastatic	IV	Male	NA	71	UBE2B	ENST00000499038: 40.67% ENST00000507277: 22.25% ENST00000506787: 16.86% ENST00000265339: 11.37%
7	Metastatic	IV	Male	NA	56	UBE2B	ENST00000507277: 46.91% ENST00000506787: 13.16% ENST00000265339: 12.6%
8	Metastatic	III	Male	Caucasian	64	UBE2B	ENST00000499038: 39.04% ENST00000507277: 26.53% ENST00000506787: 14.26% ENST00000265339: 10.59%
9	Primary	II	Male	Caucasian	84	UBE2B	ENST00000507277: 49.97% ENST00000506787: 13.82% ENST00000265339: 11.26%
10	Metastatic	IV	Male	NA	51	UBE2B	ENST00000499038: 43.41% ENST00000507277: 26.02% ENST00000506787: 10.7% ENST00000265339: 10.19%
11	Metastatic	IV	Male	NA	74	UBE2B	ENST00000499038: 38.31% ENST00000507277: 24.8% ENST00000265339: 15.27% ENST00000506787: 13.79%
12	Metastatic	IV	Male	NA	53	UBE2B	ENST00000499038: 46.74% ENST00000507277: 25.85%
13	Metastatic	II	Male	NA	50	UBE2B	ENST00000507277: 55.04% ENST00000506787: 14.5% ENST00000265339: 11.86% ENST00000499038: 10.88%
14	Metastatic	III	Male	Caucasian	63	UBE2B	ENST00000499038: 42.83% ENST00000507277: 24.37% ENST00000265339: 15.07% ENST00000506787: 11.78%
15	NA	NA	NA	NA	NA	UBE2B	ENST00000507277: 42.74% ENST00000265339: 21.21% ENST00000503080: 17.02% ENST00000499038: 10.23%
16	Primary	IV	Female	NA	61	UBE2B	ENST00000507277: 56.84% ENST00000506787: 20.5% ENST00000265339: 12.73%
17	Primary	I	Male	Caucasian	55	UBE2B	ENST00000499038: 41.44% ENST00000507277: 23.68% ENST00000265339: 18.58%
18	Metastatic	IV	Female	Caucasian	35	UBE2B	ENST00000507277: 49.37% ENST00000499038: 36.36% ENST00000265339: 14.16%
19	Metastatic	IV	Male	Caucasian	43	UBE2B	ENST00000507277: 45.05% ENST00000265339: 20.75% ENST00000503080: 14.7% ENST00000499038: 11.62%
20	Primary	III	Male	Caucasian	40	UBE2B	ENST00000499038: 58.27% ENST00000507277: 15.19% ENST00000265339: 10.35% ENST00000506787: 10.16%
21	Metastatic	III	Male	Caucasian	76	UBE2B	ENST00000499038: 47.2% ENST00000507277: 24.3% ENST00000265339: 13.93%
22	Metastatic	IV	Male	Caucasian	87	UBE2B	ENST00000507277: 55.83% ENST00000265339: 19.37% ENST00000506787: 12.65%
23	Metastatic	IV	Male	Caucasian	68	UBE2B	ENST00000499038: 43.24% ENST00000507277: 22.25% ENST00000506787: 15.65%
24	Primary	II	Male	NA	74	UBE2B	ENST00000499038: 40.05% ENST00000507277: 24.6% ENST00000506787: 14.51% ENST00000265339: 12.58%
25	Primary	IV	Male	NA	56	UBE2B	ENST00000499038: 48.37% ENST00000507277: 20.3% ENST00000265339: 12.35% ENST00000506787: 11.33%
26	Metastatic	II	Male	Caucasian	39	UBE2B	ENST00000507277: 53.09% ENST00000265339: 15.01% ENST00000506787: 14.84%
27	Primary	II	Male	Caucasian	87	UBE2B	ENST00000499038: 38.24% ENST00000265339: 21.85% ENST00000507277: 19.09%
28	Local Metast	IV	Female	Caucasian	56	UBE2B	ENST00000507277: 50.06% ENST00000503080: 17.09% ENST00000265339: 14.56%
29	Primary	II	Male	Caucasian	49	UBE2B	ENST00000499038: 53.78% ENST00000507277: 20.22% ENST00000265339: 11.91%
30	Primary	IV	Male	Caucasian	41	UBE2B	ENST00000499038: 40.95% ENST00000507277: 23.55% ENST00000506787: 13.38% ENST00000265339: 11.71%

ENST00000265339, wild type Rad6B (UBE2B)
